# Preliminary Single-Center Evaluation of the Alifax Molecular Mouse Sepsis Panel for Rapid Detection of Bloodstream Pathogens

**DOI:** 10.3390/diagnostics16152369

**Published:** 2026-07-28

**Authors:** Jay Ho Han, Jae Kwon Kim, Jung Ok Kim, Jung Suk Lee, Sungjin Jo

**Affiliations:** 1 Department of Laboratory Medicine, Uijeongbu St. Mary’s Hospital, College of Medicine, The Catholic University of Korea, Gyeonggi-do 11765, Republic of Korea; jayhohan00@gmail.com; 2 Department of Laboratory Medicine, Seoul St. Mary’s Hospital, College of Medicine, The Catholic University of Korea, Seoul 06591, Republic of Korea; jaekwon@catholic.ac.kr; 3 Department of Laboratory Medicine, Eunpyeong St. Mary’s Hospital, College of Medicine, The Catholic University of Korea, Seoul 03312, Republic of Korea; okeok91@naver.com (J.O.K.);

**Keywords:** sepsis, bloodstream infection, multiplex polymerase chain reaction, antimicrobial resistance, rapid diagnostics, blood culture

## Abstract

**Background/Objectives**: We evaluated the diagnostic performance of the Alifax Molecular Mouse (MM) sepsis panel for rapidly directly detecting pathogens and resistance genes in positive blood culture broths. **Methods**: In this prospective single-center study, 40 positive blood culture specimens were analyzed using four MM cartridges targeting Gram-positive and -negative bacteria and major antimicrobial resistance genes. The results were compared with those obtained using the routine microbiological protocol, including organism identification using the MALDI Biotyper system and antimicrobial susceptibility testing using the VITEK 2 and MicroScan WalkAway systems. Additionally, the MM panel results were compared with those obtained using the FilmArray BCID2. **Results**: Compared with the routine protocol, the MM panel achieved a positive percent agreement (PPA) of 89.5% (34/38; 95% confidence interval [CI], 75.9–95.8%) for bacterial identification, indicating moderate-to-good diagnostic performance. For antimicrobial resistance gene detection, the PPA was 92.1% (35/38; 95% CI, 79.2–97.3%). Compared with the FilmArray BCID2, the overall agreement of the MM panel was 89.4% (34/38) for bacterial identification and 84.2% (32/38) for antimicrobial resistance gene detection. The MM panel generated clinically actionable results within 1 h (mean turnaround time, 55.5 min; 95% CI, 55.1–55.9 min), substantially reducing the turnaround time compared with the conventional microbiological workflow, which requires 1.5–2 days for subculture and automated antimicrobial susceptibility testing. **Conclusions**: The MM sepsis panel enabled rapid, direct identification of bloodstream pathogens and key antimicrobial resistance markers from positive blood cultures, substantially reducing turnaround time and exhibiting its potential as an adjunct to routine microbiological workflows.

## 1. Introduction

Sepsis is one of the leading causes of mortality worldwide; therefore, early diagnosis with appropriate antimicrobial therapy is crucial for improving patient outcomes [1,2]. In clinical practice, rapidly and accurately detecting sepsis plays a pivotal role in survival and therapeutic decision-making [3]. The current gold standard for pathogen identification is blood culture, which enables microbial detection and antimicrobial susceptibility testing [4]. However, blood cultures require at least 24–72 h for identification and yielding antimicrobial susceptibility testing results, leading to significant diagnostic delays [5,6]. Moreover, these delays are associated with time-dependent increases in mortality rates [7,8], highlighting the urgent need for more rapid diagnostic methods. Molecular diagnostics enable the direct detection of trace amounts of bacterial DNA in blood, improving the speed of pathogen identification and antimicrobial resistance gene detection [9,10,11]. Recently, the Alifax Molecular Mouse (Alifax S.r.l., Polverara, Italy, MM) sepsis panel has been developed to identify multiple pathogens in blood culture-positive samples [12,13]. This panel uses polymerase chain reaction (PCR)-based technology, enabling simultaneous detection of major bacteria and resistance genes within a short turnaround time. Although multicenter evaluations have demonstrated a strong analytical performance [12], practical laboratory experience is limited. In this study, we aimed to assess the diagnostic accuracy, turnaround time, and practical applicability of an MM panel in routine clinical microbiology environments.

## 2. Materials and Methods

### 2.1. Study Design and Specimens

The blood culture-positive samples used in this study were obtained from patients who visited Eunpyeong St. Mary’s Hospital for clinical care between March and June 2025. Bloodstream infection was suspected in all cases; two or three sets of blood cultures were collected using aerobic bottles (BD BACTECPlus Aerobic/F, Becton, Dickinson and Company, Franklyn Lakes, NJ, USA) and anaerobic bottles (BD BACTEC Lytic/10 Anaerobic/F). All samples were drawn from peripheral blood, immediately transported to the laboratory, and loaded into the blood culture system (BD BACTEC FX) without delay. Blood culture bottles that exhibited positivity were immediately removed from the instrument, after which Gram staining was performed without delay, regardless of the bottle type. Blood culture-positive specimens were consecutively screened during the study period. Following Gram staining, specimens were selectively enrolled to achieve a balanced representation of Gram-positive and Gram-negative organisms, with 20 specimens included from each Gram-stain category. Duplicate isolates from the same patient were not included. Among the Gram-positive specimens, one isolate (*Bacillus licheniformis*) was inadvertently selected because it had been misinterpreted as a Gram-positive coccus on Gram staining. This specimen was subsequently excluded because the organism was not included in the target list of the Molecular Mouse (MM) sepsis panel. An additional specimen was excluded because organism identification using the routine microbiological protocol was unsuccessful. Consequently, the final analysis included 19 Gram-positive and 19 Gram-negative specimens. Organism identification and antimicrobial susceptibility testing were performed using three approaches: conventional microbiological methods (MALDI Biotyper Smart, VITEK 2 system, and MicroScan WalkAway) and two molecular diagnostic methods (Alifax sepsis panel and FilmArray Blood Culture Identification Panel 2 [BCID2]). Regardless of bottle type, samples were obtained from the first blood culture bottle that signaled positive and were used for this study. Immediately after blood culture positivity was confirmed, the MM and FilmArray BCID2 assays were performed, while conventional microbiological testing was initiated in parallel according to the standard laboratory workflow. In the routine microbiological protocol, common skin commensals isolated from only one of multiple blood culture sets were considered contaminants and were excluded from the final microbiological report. No polymicrobial blood culture specimens were identified during the study period. This study was approved by the Institutional Review Board of Eunpyeong St. Mary’s Hospital, Seoul, Republic of Korea (No. PC24DISS0108, approval date 31 October 2024), with a waiver of informed consent for using residual specimens and de-identified medical records.

### 2.2. Bacterial Identification and Susceptibility Testing

After detecting positive growth, aliquots were aseptically withdrawn from the blood culture bottle using a sterile syringe and inoculated onto 5% sheep blood agar and MacConkey agar (1 mL each) plates. The inoculated plates were incubated at 35 °C in 5% CO_2_ for 24 h. Samples that exhibited positivity in anaerobic blood culture bottles were additionally inoculated onto phenylethyl alcohol agar and incubated under anaerobic conditions (N_2_, 90%; H_2_, 5%; CO_2_, 5%) for up to 48 h.

#### 2.2.1. Alifax Molecular Mouse Sepsis Panel

Four cartridges were used: MM GRAM NEG RES (GNR24010A SI 1701.0101/L), MM GRAM NEG ID (GNI24008A SI 1701.0102/L), MM GRAM POS STAPH (GPS24003A SI 1701.0103/L), and MM GRAM POS NO STAPH (GPN24002A SI 1701.0104/L) (by Alifax s.r.l.). Details of the target organisms and antimicrobial resistance genes are provided in Supplementary Tables S1 and S2. Following the manufacturer’s instructions, 200 μL of positive blood culture broth was subjected to sequential centrifugation at 500× *g* for 1 min and 5000× *g* for 1 min. The resulting pellets were then resuspended in sterile distilled water. Depending on the cartridge type, the resuspended sample was further diluted with either distilled water or a loading solution, according to the manufacturer’s instructions. The prepared sample was then loaded onto an appropriate cartridge, inserted into the instrument, and automatically analyzed.

#### 2.2.2. Routine Protocols

For microbial identification using a MALDI Biotyper Smart mass spectrometer (Bruker Daltonics, Bremen, Germany), a 1000 μL aliquot of positive blood culture broth was mixed with 200 μL of 2% saponin and thoroughly vortexed. The mixture was centrifuged at high speed; thereafter, the supernatant was discarded. The resulting pellet was treated with 200 μL of Liquillizer and incubated for 15 min. After incubation, additional centrifugation and washing with distilled water were conducted to obtain clean pellets. The final pellet was applied to a MALDI Biotyper Smart mass spectrometer target plate (Thermo Fisher Scientific, Waltham, MA, USA) and overlaid with 0.8 μL of matrix solution. For Gram-positive cocci, an additional 0.8 μL of 70% formic acid was applied before matrix addition. The prepared spots were air-dried at room temperature (20–25 °C) before analysis. For antimicrobial susceptibility testing using the MicroScan WalkAway system (Siemens, Sacramento, CA, USA), positive blood culture samples were sub-cultured onto agar plates and incubated for 18–24 h after detecting a positive signal. Based on the resulting growth, four to five well-isolated and morphologically similar colonies were selected and suspended in 3 mL of inoculum water. The suspension was adjusted to a turbidity of 0.5 ± 0.08 McFarland using a prompt system or a photometric device. A 100 μL aliquot of the standardized suspension was then diluted into 25 mL of inoculum water containing Pluronic. The diluted suspension was loaded onto the appropriate test panel; furthermore, the instrument was operated according to the manufacturer’s instructions. The breakpoint panels contained antimicrobial concentrations in accordance with the guidelines of the Clinical and Laboratory Standards Institute [14]. Antimicrobial susceptibility testing was performed using the VITEK 2 system (bioMérieux, Marcy l’Etoile, France). For Gram-positive cocci, the P665 and ST03 cards were used, whereas for Gram-negative bacilli, the N413 and N414 cards were selected. Several well-isolated colonies were suspended in 0.45% sterile saline to prepare the inoculum, which was adjusted to a turbidity equivalent to the McFarland standard of 0.5. The prepared inoculum was loaded into a VITEK 2 cassette, and automated testing was initiated according to the manufacturer’s instructions.

#### 2.2.3. FilmArray Blood Culture Identification Panel2

The Filmarray Blood Culture Identification Panel2 (BCID; bioMérieux, Marcy l’Etoile, France) test was performed according to the manufacturer’s instructions. The pouch expiration date was verified. Hydration was performed by attaching the Hydration Injection Vial; if hydration failed, the pouch was replaced. Approximately 0.3 mL of the specimen was added to the Sample Injection Vial containing sample buffer; the vial was thoroughly mixed by capping and inverting several times. The red cap was removed, and a vial cannula was inserted into the pouch to allow vacuum-assisted specimen loading, which was visually confirmed to be successful. After successful loading, the specimen ID was recorded on the pouch label, which was inserted into the analyzer to initiate the run. The detectable organisms and antimicrobial resistance genes are summarized in Supplementary Tables S1 and S2, respectively.

### 2.3. Data Analysis

The results obtained using the MM system were compared with those of the routine microbiological protocol. Additionally, results from another molecular diagnostic platform, the FilmArray, were included for comparison. For organism identification, positive percent agreement (PPA) and the corresponding 95% confidence intervals (CIs) were calculated to evaluate the agreement between the MM system and the routine microbiological protocol. For antimicrobial resistance gene detection, positive percent agreement (PPA), negative percent agreement (NPA), overall agreement, and their corresponding 95% CIs were calculated. For organism identification, results were considered concordant when the MM system reported a broader taxonomic shared target and the corresponding species identified by the routine microbiological protocol belonged to that shared target (e.g., *Escherichia coli/Shigella* and *Enterococcus faecalis/Enterococcus faecium*). For antimicrobial resistance gene detection, results were considered concordant when the resistance gene detected by the MM system corresponded to the broader shared resistance gene target reported by the FilmArray (e.g., *vanA* vs. *vanA/B* and *mecA* vs. *mecA/C*). Confusion matrix analyses were subsequently performed to assess patterns of discordance in organism identification and antimicrobial resistance gene detection. Turnaround time was defined as the interval from assay initiation to report generation.

## 3. Results

### 3.1. Bacterial Identification Results

#### 3.1.1. Analysis of Overall Bacterial Identification Results

**Figure 1:**
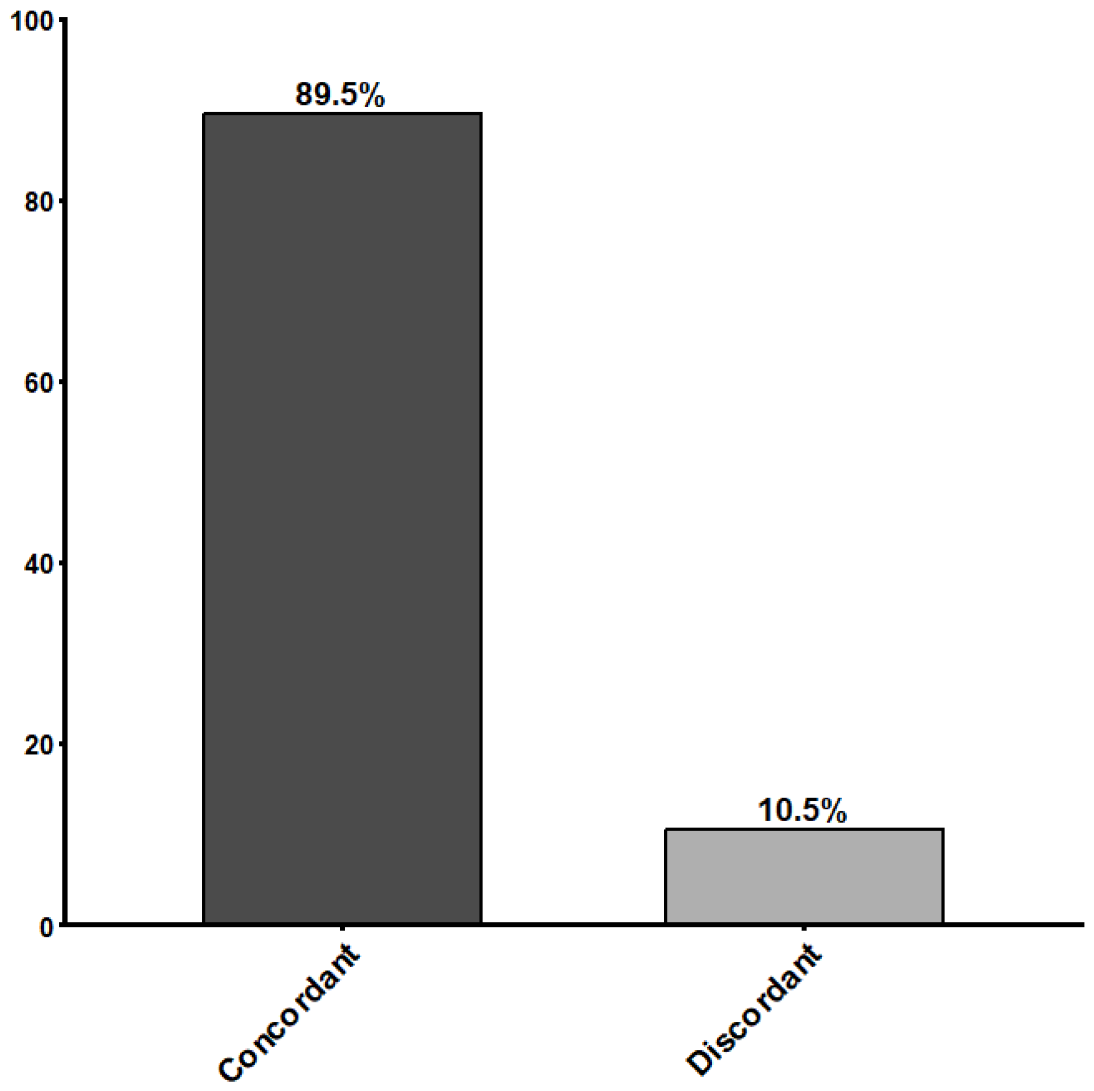
Overall agreement between the routine microbiological protocol and the Alifax Molecular Mouse Sepsis Panel for bacterial identification.

Aliquots obtained from the blood culture bottles with positive signals were subjected to Gram staining. Twenty samples exhibiting Gram-positive cocci and 20 samples exhibiting Gram-negative bacilli were selected for this study. During the study period, eligible samples that met the Gram staining criteria were enrolled. Samples exhibiting Gram-positive cocci were tested using the MM GRAM POS STAPH and MM GRAM POS NO STAPH panels, whereas samples exhibiting Gram-negative bacilli were analyzed using the MM GRAM NEG RES and MM GRAM NEG ID panels. Contrastingly, the FilmArray assay used a single kit for Gram-positive cocci and Gram-negative bacilli. Overall agreement at the genus-level bacterial identification among routine protocols and the Alifax molecular is presented in Figure 1.

Among the Gram-positive cocci, *Staphylococcus aureus* was the most frequently identified organism (*n* = 6), followed by *E. faecium* (*n* = 4), *E. faecalis* (*n* = 3), and *Staphylococcus epidermidis* (*n* = 3), whereas among Gram-negative bacilli, *E.coli* was the predominant isolate (*n* = 10), followed by *Klebsiella pneumoniae* (*n* = 4). One Gram-positive bacillus (*B. licheniformis*) was misinterpreted as a Gram-positive coccus on Gram staining. As *B. licheniformis* was not included in the MM panel, this specimen was excluded from the performance analysis. Detailed concordance data for all three methods across the 40 specimens are presented in Table 1.

At the genus level, the PPA of the MM panel was 89.5% (34/38; 95% CI, 75.9–95.8%). At the species level, the PPA decreased to 78.9% (30/38; 95% CI, 63.7–88.9%). The lower species-level PPA was attributable to four discordant cases involving *S. epidermidis*, *Staphylococcus capitis*, *E. faecium*, and *E. faecalis* (one case each). Because *S. capitis* is not included in the assay target list, species-level identification was not expected. Subgroup analysis demonstrated that the PPA of the MM panel for Gram-positive organisms was 88.9% (16/18; 95% CI, 67.2–96.9%) at the genus level and 72.2% (13/18; 95% CI, 49.1–87.5%) at the species level. Contrastingly, for Gram-negative organisms, the PPAs were identical at the genus and species levels, both 90.0% (18/20; 95% CI, 69.9–97.2%). Compared with the FilmArray BCID2, the MM panel had four discordant cases in genus-level organism identification, resulting in an overall agreement of 89.4% (34/38). The details of these discordant cases are presented in Table 1.

#### 3.1.2. Analysis of Discordant Results in Bacterial Identification

**Figure 2:**
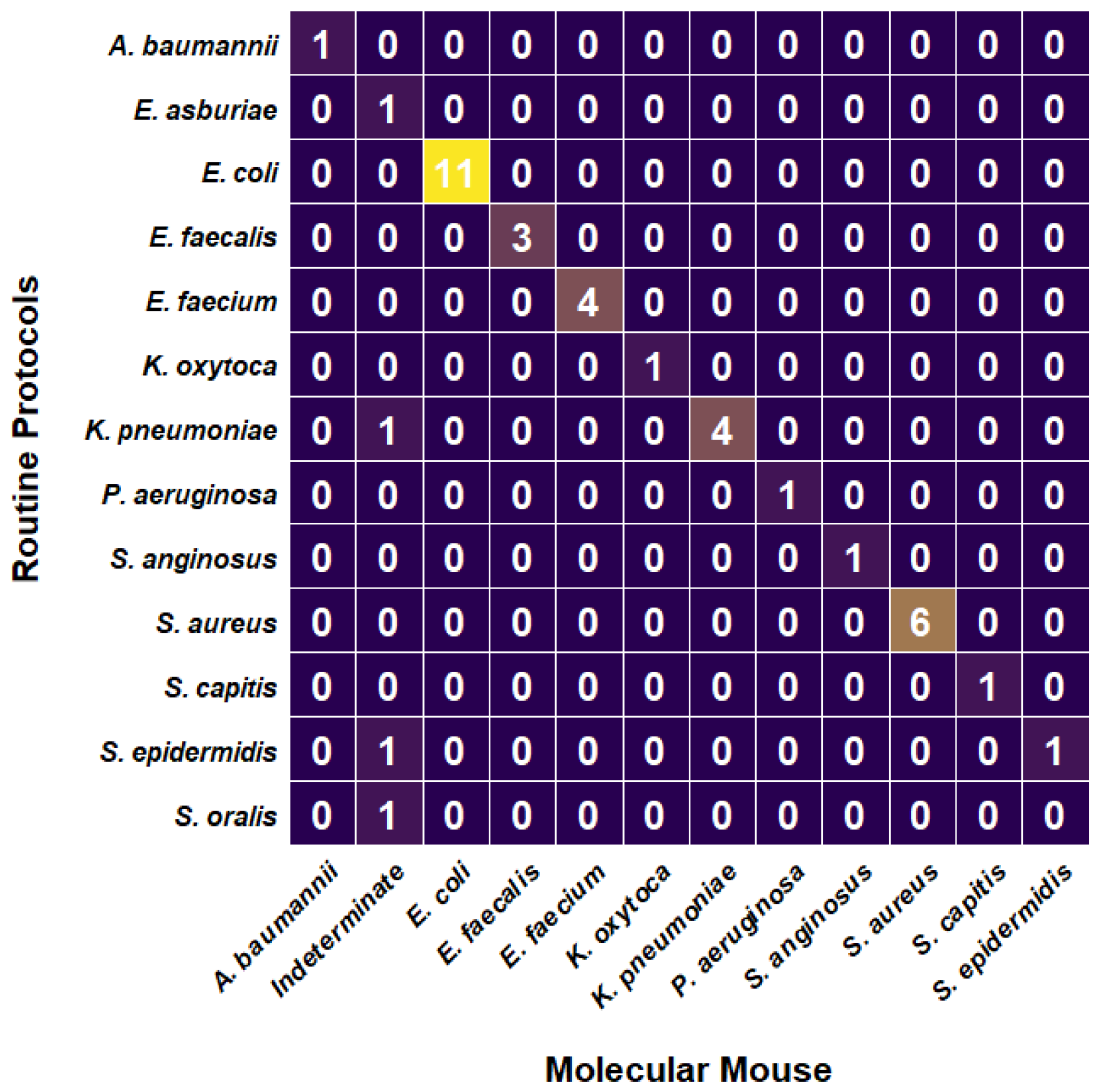
Confusion matrix comparing organism identification between routine protocols and the Alifax molecular mouse sepsis panel. Indeterminate, bacteria not identified at the species level.

At the genus level, comparison with the routine microbiological protocol identified four discordant cases involving the MM panel: *S. epidermidis*, *Streptococcus oralis*, *K. pneumoniae*, and *Enterobacter asburiae*. At the species level, four additional discordant cases were observed for the MM panel, involving *Staphylococcus capitis*, *S. epidermidis*, *E. faecium*, and *E. faecalis.* Comparison between the MM panel and the FilmArray BCID2 demonstrated four discordant cases in organism identification. All four corresponded to specimens that were not detected by the MM panel but were identified using the FilmArray BCID2. Specifically, the FilmArray BCID2 identified *K. pneumoniae* and *S. epidermidis* at the species level, whereas *S. oralis* and *E. asburiae* were identified only at the genus level, as species-level identification of these organisms is not supported by the FilmArray BCID2 panel. A comprehensive summary of discordant cases between routine protocols and the MM panel is presented in Figure 2.

### 3.2. Antimicrobial Resistance Gene Detection Results

#### 3.2.1. Analysis of Overall Antimicrobial Resistance Gene Detection Results

Among the 38 specimens included in the analysis, concordant results between the routine microbiological protocol and the MM panel were observed in 35 cases. Of these concordant cases, 19 were negative for antimicrobial resistance genes, whereas CTX-M was detected in six cases, mecA/C in five, OXA-48-like in two, and vanA/B in one. Compared with the routine microbiological protocol, the MM panel achieved an overall agreement of 92.1% (35/38; 95% CI, 79.2–97.3%), with a PPA of 85.0% (17/20; 95% CI, 63.9–94.7%) and an NPA of 100.0% (18/18; 95% CI, 82.4–100.0%) for antimicrobial resistance gene detection. In the subgroup analysis, the MM panel demonstrated an overall agreement of 89.5% (17/19; 95% CI, 68.6–97.1%), a PPA of 77.8% (7/9; 95% CI, 45.2–93.6%), and an NPA of 100.0% (10/10; 95% CI, 72.2–100.0%) for Gram-positive organisms. For Gram-negative organisms, the overall agreement was 94.7% (18/19; 95% CI, 75.4–99.1%), with a PPA of 90.9% (10/11; 95% CI, 62.2–98.3%) and an NPA of 100.0% (8/8; 95% CI, 67.5–100.0%). Compared with the FilmArray BCID2, the MM panel achieved an overall agreement of 84.2% (32/38) (Table 2).

#### 3.2.2. Analysis of Discordant Results in Antimicrobial Resistance Gene Detection

**Figure 3:**
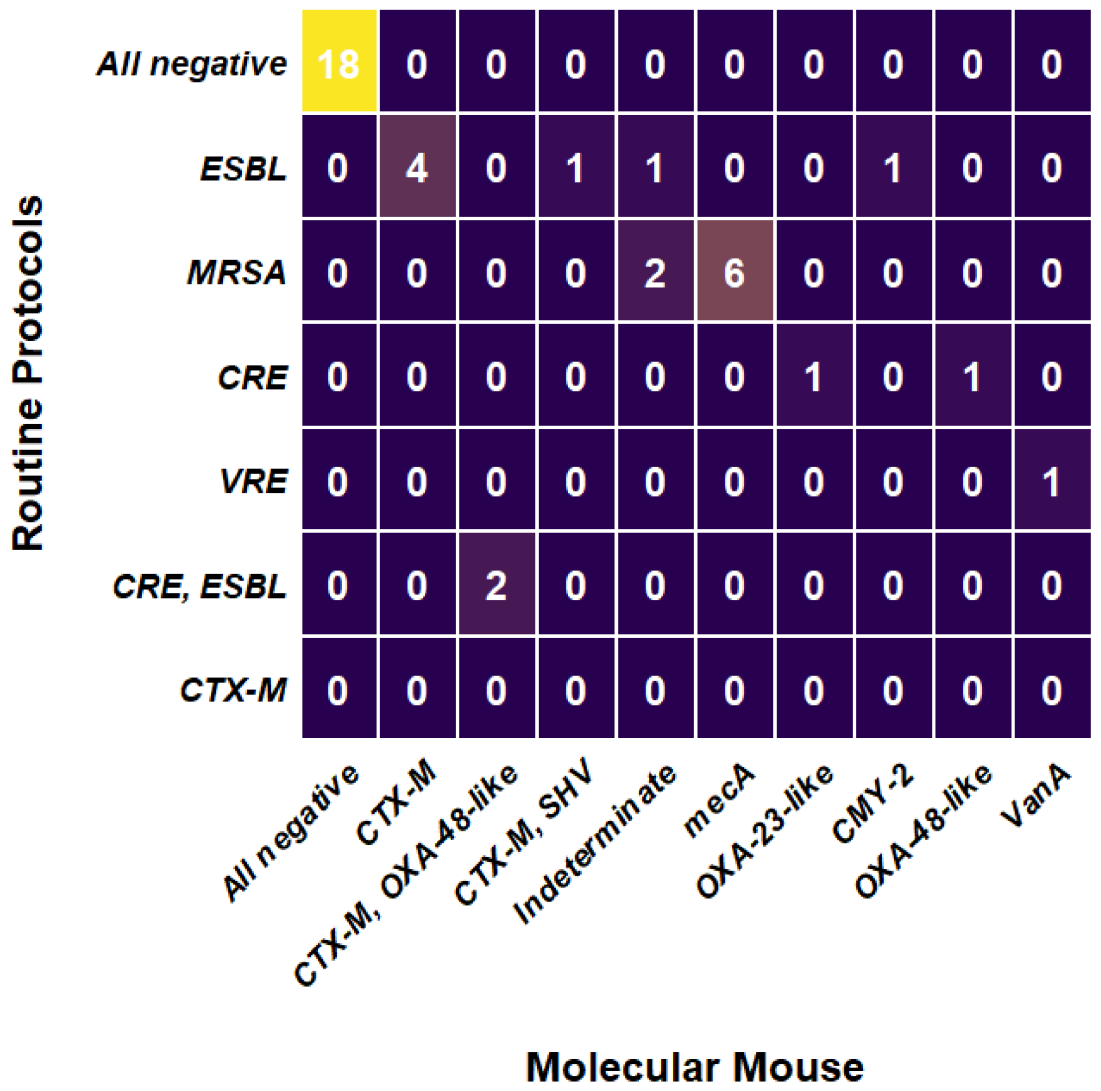
Confusion matrix comparing antimicrobial resistance gene detection between routine protocols and the Alifax molecular mouse Sepsis Panel. Indeterminate, antimicrobial resistance gene not detected.

Compared with the routine protocol, three discordant cases were observed with the MM panel, including two cases involving mecA/C and one involving CTX-M. Compared with the FilmArray BCID2, six discordant cases were identified with the MM panel. Three of these represented antimicrobial resistance genes that were not detected using the MM panel but were detected using the FilmArray BCID2, corresponding to the discordant cases identified between the MM panel and the routine protocol. In the remaining three cases, the MM panel detected antimicrobial resistance genes that were identified using the routine protocol but not using the FilmArray BCID2, including mecA, OXA-23-like, and CMY-2. A detailed summary of the discordant antimicrobial resistance gene detection results between the routine microbiological protocol and the MM panel is presented in Figure 3.

### 3.3. Turnaround Time

The MM system generated results within 1 h, substantially reducing the turnaround time compared with the 1.5–2 days required for conventional subculture-based identification and automated antimicrobial susceptibility testing. The mean turnaround time was 55.5 min (95% CI, 55.1–55.9 min).

## 4. Discussion

The sustained increase in the rate of antimicrobial resistance (AMR) is a global public health threat with substantial clinical and economic consequences [15,16]. The increase in AMR leads to increased mortality and morbidity, longer stays in hospitals, and increased health care costs. Bloodstream infections are common in hospitals [17,18]; rapid identification of the causative pathogens is a challenge in sepsis management. Molecular diagnostic platforms have become valuable adjuncts to traditional culture-based techniques, significantly shortening the time required for pathogen identification and resistance gene detection, facilitating early administration of more targeted therapy [19,20,21]. Of the 40 specimens initially enrolled, 1 Gram-negative bacillus specimen was excluded because it was not identified using the routine microbiological protocol. Another specimen, which contained *B. licheniformis*, had been misclassified as Gram-positive cocci using Gram staining and was excluded because *B. licheniformis* is not included in the MM GRAM POS NO STAPH panel. Consequently, the final analysis was performed using 38 specimens. At the genus level, the PPA of the MM panel relative to routine protocols was 89.5% (34/38; 95% CI, 75.9–95.8%), indicating a moderate-to-good concordance. Four discordant cases were identified, including *S. oralis*, *K. pneumonia*, *E. asburiae*, and *S. epidermidis*. At the genus level, four discordant cases were identified, with an additional four discordant cases at the species level. At the species level, *E. faecium* and *E. faecalis* were identified only as *Enterococcus* spp. using the MM panel. As other *E. faecium* (*n* = 3) and *E. faecalis* (*n* = 2) isolates were correctly identified to the species level using the MM panel, these discrepancies are more likely explained by preprocessing-related factors, including low bacterial load or sample loss during manual processing, rather than primer specificity. The sequential centrifugation steps required for MM panel preprocessing may contribute to such variability. Compared with the FilmArray BCID2, the MM panel yielded four discordant identification results, all of which were false-negative relative to the routine microbiological protocol, involving *K. pneumoniae* and *S. epidermidis* at the species level and *S. oralis* and *E. asburiae* at the genus level. Given that the FilmArray BCID2, which does not require manual preprocessing or centrifugation, correctly identified these organisms in the same specimens, the discordant results are more likely attributable to sample preprocessing than to differences in assay performance. This interpretation is further supported by large-scale studies reporting 100% sensitivity and specificity for these targets [22]. For antimicrobial resistance gene detection, the MM panel achieved a PPA of 92.1% (35/38; 95% CI, 79.2–97.3%) compared with that of the routine protocol, indicating good diagnostic performance. Three discordant cases were observed, including two involving mecA/C and one involving CTX-M. Compared with the FilmArray BCID2, six discordant cases were identified. Three were identical to the discordant cases observed with the routine protocol, whereas in the remaining three cases, the MM panel detected mecA, OXA-23-like, and CMY-2, all of which were concordant with the routine protocol but not detected by the FilmArray BCID2. Most discordant specimens were derived from anaerobic blood culture bottles, suggesting that specimen type may have influenced assay performance. Among the four discordant cases, three (75.0%) at the genus level (*S. oralis*, *S. epidermidis*, and *K. pneumoniae*) and three (75.0%) at the species level (*S. epidermidis*, *E. faecalis*, and *E. faecium*) were obtained from anaerobic blood culture bottles. Furthermore, all three discordant cases (100%) involving antimicrobial resistance gene detection, including two cases of mecA and one case of CTX-M, were derived from anaerobic blood culture bottles. One possible explanation is reduced DNA recovery associated with the resin-containing, high-viscosity anaerobic culture medium, which may interfere with nucleic acid extraction. Additionally, bacterial lysis occurring during prolonged incubation under anaerobic conditions could decrease the amount of recoverable target DNA. Although these factors were not directly evaluated in the present study, they may have contributed to the observed false-negative results. Future studies incorporating optimized sample-preprocessing methods, such as enzymatic lysis enhancement or host DNA depletion, may improve analytical sensitivity in anaerobic blood culture specimens. For the discordant cases derived from aerobic blood culture bottles, procedural factors associated with pellet preparation and sample loading into the Alifax cartridge wells may have contributed to the false-negative results. Given the manual preprocessing steps required before analysis, variability in specimen handling could potentially affect DNA recovery and subsequent target detection. Furthermore, in specimens that were not identified using either the MM panel or MALDI Biotyper Smart mass spectrometer but were detected using FilmArray BCID2, partial bacterial loss during centrifugation and pellet-processing steps may represent another possible source of discordance. Accordingly, although the MM panel demonstrated a genus-level PPA of 89.5% (34/38; 95% CI, 75.9–95.8%), the PPA decreased to 78.9% (30/38; 95% CI, 63.7–88.9%) at the species level, indicating reduced clinical performance for species-level identification. These findings suggest that preanalytical variables, particularly manual preprocessing steps, may adversely affect assay performance. Therefore, future studies are warranted to evaluate the impact of these preanalytical variables and to optimize sample preparation procedures. In particular, refinement of preprocessing strategies, including enzymatic lysis-based cell disruption and the development of specimen-specific protocols for anaerobic blood culture bottles, may improve nucleic acid recovery and reduce false-negative results. Conversely, in three cases in which the FilmArray BCID2 failed to detect resistance genes, the MM panel successfully identified OXA-23-like in *A. baumannii*, OXA-23-like and CMY-2 in *E. coli*, and *mec*A in *S. capitis*. The results were obtained within 1 h, which was markedly faster than the conventional workflow that required positive blood culture confirmation, subculture, and subsequent antimicrobial susceptibility testing using instruments such as VITEK 2, typically taking 1.5–2 days. Rapid identification and determination of susceptibility enable early clinical interventions in bloodstream infections. This capability could support antimicrobial stewardship programs, particularly in intensive care unit settings where timely optimization of antimicrobial treatment is critical. Since the MM system has already been evaluated in large multicenter studies, the aim of the present study was to assess its performance in practical single-center settings. The relatively small sample size is an important limitation of this study, as it restricted a comprehensive assessment of the full spectrum of target pathogens. Consequently, the calculated sensitivity and PPA should be interpreted with caution, as the limited number of specimens may have reduced the precision of these estimates and increased their susceptibility to random error. Additionally, 16S rRNA gene Sanger sequencing was not performed for all specimens because of practical and logistical constraints. Therefore, the routine microbiological workflow, including identification using the MALDI Biotyper Smart mass spectrometer and conventional antimicrobial susceptibility testing, was used as the reference standard for performance evaluation. Finally, the MM panel requires manual preprocessing, including centrifugation and pellet recovery, which may introduce preanalytical variability through DNA loss, contamination, or reduced bacterial load. However, the lack of repeat testing and internal control data limited our ability to directly assess the impact of these factors on assay performance. Despite these constraints, the MM platform could ensure rapidity, flexibility, and ease of operation, providing clinically relevant results from positive blood cultures in approximately 1 h. Its compact format and intuitive interface render it well-suited for decentralized testing and seamless integration into routine clinical microbiology workflows.

## 5. Conclusions

The Alifax MM sepsis panel enabled rapid and direct detection of bloodstream pathogens and antimicrobial resistance markers from positive blood cultures. Combined with a turnaround time of less than 1 h, the system is a valuable adjunct to conventional microbiological workflows. Further optimization of the manual preprocessing procedure may improve species-level identification performance and enhance the overall clinical utility of the assay.

## Figures and Tables

**Table 1:** The overall results of bacteria identification.

Specimen No.	Routine Protocols	Molecular Mouse	FilmArray BCID Panel	Blood Culture Bottle Type
1	*Escherichia coli*	*E. coli/Shigella* spp.	*E. coli*	Plus Aerobic/F
2	*Klebsiella pneumoniae*	*K. pneumoniae*	*K. pneumonia* *e*	Plus Aerobic/F
3	*E. coli*	*E. coli/Shigella* spp.	*E. coli*	Plus Aerobic/F
4	*S. aureus*	*S. aureus*	*S. aureus*	Plus Aerobic/F
5	*S. aureus*	*S. aureus*	*S. aureus*	Plus Aerobic/F
6	*P. aeruginosa*	*P. aeruginosa*	*P. aeruginosa*	Plus Aerobic/F
7	*E. coli*	*E. coli/Shigella* spp.	*E. coli*	Plus Aerobic/F
8	*A. baumannii*	*A. baumannii*	*A. baumannii*	Plus Aerobic/F
9	*K. pneumoniae*	*K. pneumoniae*	*K. pneumoniae*	Lytic/10 Anaerobic/F
10	*E. coli*	*E. coli/Shigella* spp.	*E. coli*	Plus Aerobic/F
11	*K. pneumoniae*	*K. pneumoniae*	*K. pneumoniae*	Plus Aerobic/F
12	*K. oxytoca*	*K. oxytoca*	*K. oxytoca*	Lytic/10 Anaerobic/F
13	*S. aureus*	*S. aureus*	*S. aureus*	Lytic/10 Anaerobic/F
14	*E. faecalis*	*E. faecalis*	*E. faecalis*	Lytic/10 Anaerobic/F
15	*E. faecium*	*E. faecium*	*E. faecium*	Lytic/10 Anaerobic/F
16	*E. faecalis*	*E. faecalis*	*E. faecalis*	Lytic/10 Anaerobic/F
17	*E. faecium*	***Enterococcus* spp**.	*E. faecium*	Lytic/10 **Anaerobic**/F
18	*E. coli*	*E. coli/Shigella* sp.	*E. coli*	Lytic/10 Anaerobic/F
19	*S. capitis/S. epidermidis*	***Staphylococcus*** **spp.**	*S. epidermidis*	Lytic/10 **Anaerobic**/F
20	*B. licheniformis*	**N/A** ^a^	**N/A** ^a^	Plus Aerobic/F
21	*S. oralis*	**N/A** ^b^	***Streptococcus*** **spp.**	Lytic/10 **Anaerobic**/F
22	*S. capitis*	***Staphylococcus*** **spp.**	***Staphylococcus*** **spp.**	Plus Aerobic/F
23	*K. pneumoniae*	**N/A** ^b^	*K. pneumoniae*	Lytic/10 **Anaerobic**/F
24	*E. coli*	*E. coli/Shigella* sp.	*E. coli*	Lytic/10 Anaerobic/F
25	*S. aureus*	*S. aureus*	*S. aureus*	Lytic/10 Anaerobic/F
26	*E. faecalis*	***Enterococcus*** **spp.**	*E. faecalis/E. faecium*	Lytic/10 **Anaerobic**/F
27	*S. aureus*	*S. aureus*	*S. aureus*	Plus Aerobic/F
28	*S. anginosus*	*S. anginosus*	***Streptococcus*** **spp.**	Lytic/10 Anaerobic/F
29	*S. aureus*	*S. aureus*	*S. aureus*	Lytic/10 Anaerobic/F
30	*S. epidermidis*	**N/A** ^b^	*S. epidermidis*	Lytic/10 **Anaerobic**/F
31	**N/A** ^b^	**N/A** ^b^	*E. faecium*	Plus Aerobic/F
32	*E. faecium*	*E. faecium*	*E. faecium*	Plus Aerobic/F
33	*E. coli*	*E. coli/Shigella* sp.	*E. coli*	Lytic/10 Anaerobic/F
34	*E. faecium*	*E. faecium*	*E. faecium*	Plus Aerobic/F
35	*E. asburiae*	**N/A** ^b^	***Enterobacterales***	Plus Aerobic/F
36	*E. coli*	*E. coli/Shigella* sp.	*E. coli*	Plus Aerobic/F
37	***Escherichia*** **spp.**	*E. coli/Shigella* sp.	*E. coli*	Plus Aerobic/F
38	*E. coli*	*E. coli/Shigella* sp.	*E. coli*	Lytic/10 Anaerobic/F
39	*E. coli*	*E. coli/Shigella* sp.	*E. coli*	Plus Aerobic/F
40	***K.*** **spp.**	*K. pneumoniae*	*K. pneumoniae*	Plus Aerobic/F

^a^: organism not included in the panel, ^b^: despite successful assay performance, the result was negative for the target pathogen.

**Table 2:** Overall antimicrobial gene results.

Specimen No.	Identification	Phenotype	Molecular Mouse	FilmArray BCID Panel
1	*E. coli*	ESBL	CTX-M-1/9 group, OXA-23(LOW)	CTX-M
2	*K. pneumoniae*	ESBL	SHV ESBL, CTX-M-1/9 group, OXA-48-like	CTX-M, OXA-48-like
3	*E. coli*	N/A	N/A ^a^	N/A ^a^
4	*S. aureus*	N/A	N/A ^a^	N/A ^a^
5	*S. aureus*	MRSA	*mecA*	*mecA/C* and *MREJ(MRSA)*
6	*P. aeruginosa*	N/A	N/A ^a^	N/A ^a^
7	*E. coli*	ESBL	CTX-M-1/9 group	CTX-M
8	*A. baumannii*	CRE	**OXA-23-like**	**N/A** ^b^
9	*K. pneumoniae*	CRE	CTX-M-1/9 group, OXA-48-like	CTX-M, OXA-48-like
10	*E. coli*	N/A	N/A ^a^	N/A ^a^
11	*K. pneumoniae*	CRE	SHV, OXA-48-like	OXA-48-like
12	*K. oxytoca*	ESBL	CTX-M-1/9 group	CTX-M
13	*S. aureus*	**MRSA**	**N/A** ^b^	***mecA/C*** **and** ***MREJ(MRSA)***
14	*E. faecalis*	N/A	N/A ^a^	N/A ^a^
15	*E. faecium*	N/A	N/A ^a^	N/A ^a^
16	*E. faecalis*	N/A	N/A ^a^	N/A ^a^
17	*E. faecium*	N/A	N/A ^a^	N/A ^a^
18	*E. coli*	ESBL	**OXA-23-like, CMY-2**	**N/A** ^b^
19	*S. capitis/* *S. epidermidis*	MRSA	*mecA*	*mecA/C*
20	*B. licheniformis*	-	N/A ^a^	N/A ^a^
21	*S. oralis*	-	N/A ^a^	N/A ^a^
22	*S. capitis*	MRSA	***mecA***	**N/A** ^b^
23	*K. pneumonia*	N/A	N/A ^a^	N/A ^a^
24	*E. coli*	ESBL	CTX-M-1/9 group	CTX-M
25	*S. aureus*	**MRSA**	**N/A** ^b^	***mecA/C*** **and** ***MREJ(MRSA)***
26	*E. faecalis*	N/A	N/A ^a^	N/A ^a^
27	*S. aureus*	MRSA	*mecA*	*mecA/C* and *MREJ(MRSA)*
28	*S. anginosus*	N/A	N/A ^a^	N/A ^a^
29	*S. aureus*	MRSA	*mecA*	*mecA/C* and *MREJ(MRSA)*
30	*S. epidermidis*	MRSA	*mecA*	*mecA/C*
31	*E. faecium*		**N/A** ^b^	***VanA/B***
32	*E. faecium*	VRE	*VanA*	*VanA/B*
33	*E. coli*	N/A	N/A ^a^	N/A ^a^
34	*E. faecium*	N/A	N/A ^a^	N/A ^a^
35	*E. asburiae*	N/A	N/A ^a^	N/A ^a^
36	*E. coli*	N/A	N/A ^a^	N/A ^a^
37	*Escherichia* spp.	N/A	N/A ^a^	N/A ^a^
38	*E. coli*	**ESBL**	**N/A** ^b^	**CTX-M**
39	*E. coli*	N/A	N/A ^a^	N/A ^a^
40	*K.* spp.	CRE	CTX-M-1/9 group, OXA-48-like	OXA-48-like

^a^: no gene detected, ^b^: despite successful assay performance, the result was negative for the target pathogen.

## Data Availability

The data presented in this study are available upon request from the corresponding author.

## Supplementary Materials

The following supporting information can be downloaded at: https://www.mdpi.com/article/10.3390/diagnostics16152369/s1, Tables S1. Comparison of detectable organisms between the Alifax molecular mouse sepsis panel and FilmArray blood culture Identification Panel2; Tables S2. Comparison of detectable antimicrobial resistance genes between the Alifax molecular mouse sepsis panel and FilmArray blood culture Identification Panel2.
