# Supplementary material for: Preliminary Single-Center Evaluation of the Alifax Molecular Mouse Sepsis Panel for Rapid Detection of Bloodstream Pathogens
Source: Diagnostics (Basel). 2026 Jul 28;16(15):2369. doi: 10.3390/diagnostics16152369 (PMC13465562; doi:10.3390/diagnostics16152369)
Supplement: Supplementary file 1 [file diagnostics-16-02369-s001.zip › diagnostics-4335495-supplementary.pdf]

Supplementary Tables

Tables S1. Comparison of detectable organisms between the Alifax molecular mouse sepsis panel and FilmArray blood culture Identification Panel2.

Category	Alifax molecular mouse sepsis panel	FilmArray Blood Culture Identification Panel2
Gram-positive bacteria	*Staphylococcus aureus*[a]	*Staphylococcus aureus*
	Staphylococcus epidermidis[a]	*Staphylococcus epidermidis*
	Staphylococcus hominis[a]	*S. lugdunensis*
	Staphylococcus spp.[a]	Limited
	Staphylococcus simulans[a]	Limited
	Staphylococcus haemolyticus[a]	Limited
	Staphylococcus sciuri[a]	Limited
	Staphylococcus xylosus[a]	Limited
	Staphylococcus lugdunensis[a]	Limited
	Staphylococcus saprophyticus[a]	Limited
	Streptococcus spp.[b]	*Streptococcus spp*
	Streptococcus agalactiae[b]	*Streptococcus agalactiae*
	Streptococcus anginosus[b]	Limited
	Streptococcus pneumoniae[b]	*Streptococcus pneumoniae*
	Streptococcus pyogenes[b]	*Streptococcus pyogenes*
	Enterococcus spp.[b]	*Enterococcus spp.*
	Enterococcus faecalis[b]	*Enterococcus faecalis*
	Enterococcus faecium[b]	*Enterococcus faecium*
	Listeria monocytogenes[b]	*Listeria monocytogenes*
	Bacillus subtilis[b]	Limited
Gram-negative bacteria	*Klebsiella oxytoca*[c]	*Klebsiella oxytoca*

	Klebsiella pneumoniae[c]	*Klebsiella pneumoniae group*
	Klebsiella aerogenes[c]	*Klebsiella aerogenes*
	Escherichia Coli/Shigella spp.[c]	*Escherichia coli*
	Salmonella typhi[c]	*Salmonella group*
	Enterobacter cloacae[c]	*Enterobacter cloacae complex*
	Enterobacteriaceae[c]	*Acinetobacter baumannii*
	Pseudomonas aeruginosa[c]	*Pseudomonas aeruginosa*
	Haemophilus influenzae[c]	*Haemophilus influenzae*
	Acinetobacter baumannii[c]	*Acinetobacter baumannii-calcoaceticus complex*
	Neisseria meningitidis[c]	*Neisseria meningitidis*
	Stenotrophomonas maltophilia[c]	*Stenotrophomonas maltophilia*
	Serratia marcescens[c]	*Serratia marcescens*
	Proteus mirabilis[c]	Limited
	Proteus spp.[c]	*Proteus spp.*
	Limited	*Bacteroides fragilis*

[a] MM GRAM POS STAPH (GPS24003A SI 1701.0103/L).

[b] MM GRAM POS NO STAPH (GPN24002A SI 1701.0104/L).

[c] MM GRAM NEG ID (GNI24008A SI 1701.0102/L).

Table S2. Comparison of detectable antimicrobial resistance genes between the Alifax molecular mouse sepsis panel and FilmArray blood culture Identification Panel2.

**Category**	**Alifax sepsis panel**	**FilmArray Blood Culture Identification Panel2**
Methicillin resistance	*mecA*[a], *mecC*[a], *SCCmec-orfX*[a]	*mecA/C + MREJ*
Vancomycin resistance	*vanA*[a,b], *vanB*[b], *vanC1*[b], *van C2/3*[b]	*vanA, vanB*
AmpC / β-lactamase	*CMY2*[c]	Limited

	SHV[c]	Limited
ESBL	*CTX-M (1/9 group, 2/8 group)*[c]	*CTX-M*
	SHV ESBL[c]	Limited
Carbapenemase	*KPC*[c]	*KPC*
	NDM[c]	*NDM*
	VIM[c]	*VIM*
	OXA-48-like[c]	*OXA-48-like*
	OXA-23-like[c]	Limited
	IMP[c]	*IMP*
Colistin	*mcr-1, mcr-2*[c]	*mcr-1*

[a] MM GRAM POS STAPH (GPS24003A SI 1701.0103/L)

[b] MM GRAM POS NO STAPH (GPN24002A SI 1701.0104/L)

[c] MM GRAM NEG RES (GNR24010A SI 1701.0101/L)
